# Potential alternative drug treatment for bone giant cell tumor

**DOI:** 10.3389/fcell.2023.1193217

**Published:** 2023-06-13

**Authors:** Zhangxin Chen, Cong Zhang, Haisen Hong, Wenbin Xu, Mo Sha, Zhenqi Ding

**Affiliations:** ^1^ Department of Orthopedics, The 909th Hospital, School of Medicine, Xiamen University, Zhangzhou, China; ^2^ School of Medicine, Xiamen University, Xiamen, China; ^3^ Department of Orthopedics, The First Affiliated Hospital of Xiamen University, Xiamen, China

**Keywords:** bone giant cell tumor, fracture healing, potential genes, potential drugs, drug treatment

## Abstract

**Background:** Bone giant cell tumor (BGCT) is one of the world’s major disease types of locally aggressive bone tumors. In recent years, denosumab treatment has been introduced before curettage surgery. However, the current therapeutic was practical only sometimes, given the local recurrence effects after discontinuation of denosumab. Due to the complex nature of BGCT, this study aims to use bioinformatics to identify potential genes and drugs associated with BGCT.

**Methods:** The genes that integrate BGCT and fracture healing were determined by text mining. The gene was obtained from the pubmed2ensembl website. We filtered out common genes for the function, and signal pathway enrichment analyses were implemented. The protein–protein interaction (PPI) networks and the hub genes were screened by MCODE built-in Cytoscape software. Lastly, the confirmed genes were queried in the Drug Gene Interaction Database to determine potential genes and drugs.

**Results:** Our study finally identified 123 common specific genes in bone giant cell tumors and fracture healing text mining concepts. The GO enrichment analysis finally analyzed 115 characteristic genes in BP, CC, and MF. We selected 10 KEGG pathways and identified 68 characteristic genes. We performed protein–protein interaction analysis (PPI) on 68 selected genes and finally identified seven central genes. In this study, these seven genes were substituted into drug–gene interactions, and there were 15 antineoplastic drugs, 1 anti-involving drug, and 1 anti-influenza drug.

**Conclusion:** The 7 genes (including *ANGPT2*, *COL1A1*, *COL1A2*, *CTSK*, *FGFR1*, *NTRK2*, and *PDGFB*) and 17 drugs, which have not been used in BGCT, but 6 of them approved by the FDA for other diseases, could be potential genes and drugs, respectively, to improve BGCT treatment. In addition, the correlation study and analysis of potential drugs through genes provide great opportunities to promote the repositioning of drugs and the study of pharmacology in the pharmaceutical industry.

## Introduction

Bone giant cell tumor (BGCT) is a locally aggressive benign bone tumor that is most commonly found in the distal femur and proximal tibia. BGCT accounts for 3%∼5% of all primary bone tumors. The peak onset of BGCT occurs between the ages of 30 and 40. Metastasis of BGCT is rarer than that of other malignancies (Raskin et al., 2013; Montgomery et al., 2019).

Surgery is the primary treatment for bone giant cell tumors. Surgical resection methods include intralesional curettage and motorized reaming or *en bloc* resection. Autologous and allogeneic cells, and bone cement filled the bone defect (Traub et al., 2016). Local recurrence rates after curettage were about 30% (Balke et al., 2008; Becker et al., 2008; Kivioja et al., 2008; Algawahmed et al., 2010). Surgery-related local adjuncts are currently used to reduce the rate of local recurrence: phenol, hydrogen peroxide, liquid nitrogen, regional chemotherapy, electrocoagulation, and heat generated by acrylic cement; however, no real benefit has been documented (Algawahmed et al., 2010).

Denosumab is the most classic systemic treatment drug for BGCT and has been approved by the FDA since 2013 for patients with unresectable tumors or those who have a score of 8 on the skeletal maturity index. BGCT contains two significant cell populations: reactive multinucleated osteoclast-like giant cells expressing nuclear factor KAPpa-B (RANK) receptor activator and neoplastic mononuclear stromal cells expressing the RANK ligand (RANKL). Denosumab is a complete human monoclonal antibody that inhibits the receptor activator of the nuclear factor kappa beta ligand; denosumab prevents osteolysis by inhibiting the recruitment of reactive osteoclast-like giant cells bytumor stromal cells (Thomas et al., 2010). Related meta-analysis showed that denosumab could reduce tumor mass and even decorticate the edge of the lesion to reduce pain and surgical morbidity. However, the preoperative use of denosumab remains controversial (Sano et al., 2020). According to a related systematic review, denosumab treatment may even be associated with an increase in the proportion of patients experiencing local recurrence (Tsukamoto et al., 2020).

It often takes about 10 years for a new drug to go from laboratory development to market. During this period, the investment and workforce required to conduct clinical trials are staggering, and the results of such investments are unpredictable. Therefore, searching for a new range of treatments for existing drugs may be another feasible and effective way to solve the problem of new drug discovery. Currently, increasing attention is being paid to text mining based on the medical and biological literature worldwide. In recent years, the utilization rate of FDA-approved vaccines and drugs has been as high as 30%, which also shows the great potential of text mining. Case in point: propranolol, a form of definitive treatment for coronary heart disease and high blood pressure, has recently been found for osteoporosis and melanoma.

**FIGURE 1:**
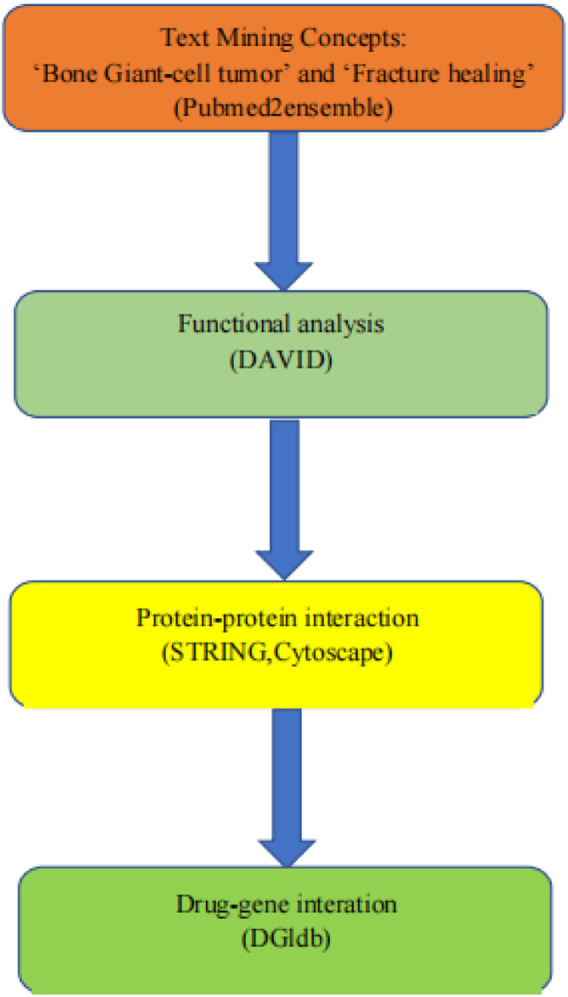
Overall data mining strategy. Text mining was used to identify genes associated with the concepts of BGCT and BF using pubmed2ensemble. Extracted genes were then analyzed for their function and gene ontology using DAVID. Further enrichment was obtained by molecular network analysis using STRING and Cytoscape. The final enriched gene list was then used to determine interactions with known drugs using the Drug Gene Interaction Database.

The genes that integrate BGCT and fracture healing were determined by text mining. The gene was obtained on the pubmed2ensembl website. We filtered out common genes for the function and genes implemented in signal pathway enrichment analyses. The protein–protein interaction (PPI) networks and the hub genes were screened by MCODE built-in Cytoscape software. Lastly, the confirmed genes were queried in the Drug Gene Interaction Database to determine potential genes and drugs. Figure 1 shows the workflow of this study.

## Materials and methods

### Text mining

The web-based service pubmed2ensembl (http://pubmed2ensembl.ls.manchester.ac.uk/) was used to perform text mining. The link extension to the BioMart system links over 2,000,000 articles in PubMed to nearly 150,000 genes in Ensembl from 50 species (Baran et al., 2011). Users allow text-based queries to be performed against PubMed and PubMed Central documents in conjunction with constraints on genomic features. We performed two questions for producing two gene lists in the study, one with the idea of a bone giant cell tumor (BGCT) and the other with the concept of fracture healing (FH). We filtered common genes and then used them to proceed to the next steps.

### GO enrichment analysis

The study used the DAVID database (http://david.ncifcrf.gov/summary.jsp) for a GO enrichment analysis of the shared genes of the BGCT and FH intersection, including biological process (BP), cellular component (CC), and molecular function (MF). The GO enrichment analysis selected all the top 8 genes with the lowest *p*-values. DAVID grouping of such identifiers improves the cross-reference capability, particularly across the NCBI and UniProt systems, enabling more than 40 publicly available functional annotation sources to be comprehensively integrated (Dennis et al., 2003).

### Kyoto Encyclopedia of Genes and Genomes (KEGG) pathway analysis

Later, we centralized 10 genes with the lowest *p*-values by the DAVID Kyoto Encyclopedia of Genes and Genomes (KEGG) pathways that contained the involved genes. Characteristic genes shared by the GO enrichment and KEGG pathway analyses were selected and included in subsequent analyses (Pathan et al., 2017).

### Protein–protein interaction (PPI) network and gene module analysis

The study uses the Search Tool for the Retrieval of Interacting Genes (STRING) database (http://string-db.org), which is an open access database designed to evaluate the protein–protein interaction (PPI) messages of common genes. The STRING (version 10.5) database integrates text mining in PubMed and covers 9.6 million proteins originating from 2031 organisms (Szklarczyk et al., 2015). At first, we uploaded and mapped the list of the GO enrichment analysis and KEGG pathways involved in genes to the STRING website. Then, PPIs of the shared genes with a minimum required interaction score >0.9 (highest confidence) hid the disconnected nodes in the network. After that, Cytoscape software constructed PPI networks. The significant gene modules of the PPI networks were applied to pick out in Cytoscape with the Molecular Complex Detection (MCODE). The parameters were set as follows: the degree cutoff >2, K-scores >2, and node score cutoff >0.2. Finally, we selected the most significant gene modules from the PPI networks for further validation analyses.

### Quantitative real-time (qRT)-PCR array validation

To validate the findings of the bioinformatics analysis, lesion tissue from patients was harvested for qRT-PCR validation between the BGCT group (*n* = 6) and the control group (*n* = 6). The use of verbal consent was approved by the Southeast Hospital of Xiamen University, and verbal consent was obtained from each participant. Total RNA was extracted from tissue lesions using the TRIzol reagent (Yisheng, Shanghai, China). RNA samples were reverse transcribed to cDNA using the First Strand cDNA Synthesis Kit (Yisheng, China), according to the manufacturer’s instructions, with set conditions of 25°C/5 min, 42°C/30 min, and 85°C/5 min. After completion of reverse transcription, the system was diluted 10-fold because the reaction conditions included pre-denaturation at 95°C for 5 min, and cycling at 95°C/10 s and 60°C/30 s (40 cycles). The primers were validated by NABI blast and then synthesized by Xiamen BoRui Biotechnology Co. The relative expression of mRNA was calculated using the 2^−ΔΔCT^ method. p< 0.05 was considered a statistically significant difference. The primer sequences are shown in Table 1.

**TABLE 1:** List of primer sequences used for quantitative real-time PCR.

Gene	Sequences	
	Forward	Reverse
*ANGPT2*	AAC​TTT​CGG​AAG​AGC​ATG​GAC	CGA​GTC​ATC​GTA​TTC​GAG​CGG
*COL1A1*	GAG​GGC​CAA​GAC​GAA​GAC​ATC	CAG​ATC​ACG​TCA​TCG​CAC​AAC
*COL1A2*	GGC​CCT​CAA​GGT​TTC​CAA​GG	CAC​CCT​GTG​GTC​CAA​CAA​CTC
*CTSK*	ACT​CAA​AGT​ACC​CCT​GTC​TCA​T	CCA​CAG​AGC​TAA​AAG​CCC​AAC
*FGFR1*	CCC​GTA​GCT​CCA​TAT​TGG​ACA	TTT​GCC​ATT​TTT​CAA​CCA​GCG
*NTRK2*	TCG​TGG​CAT​TTC​CGA​GAT​TGG	TCG​TCA​GTT​TGT​TTC​GGG​TAA​A
*PDGFB*	CTC​GAT​CCG​CTC​CTT​TGA​TGA	CGT​TGG​TGC​GGT​CTA​TGA​G

*QPCR*-related genes include *ANGPT2*, *COL1A1*, *COL1A2*, *CTSK*, *FGFR1*, *NTRK2*, and *PDGFB*.

### Drug–gene interactions

The final list of genes was used as the potential targets in a search for existing drugs or small organic compounds. The Drug–Gene Interaction Database (DGIdb, www.dgidb.org) is a web resource that consolidates disparate data sources describing drug–gene interactions and gene druggability (Wagner et al., 2016). It provides an intuitive graphical user interface and a documented application programming interface (API) for querying these data. The STITCH database (http://stitch.embl.de/) integrates these disparate data sources for 430,000 chemicals into a single, easy-to-use resource (Szklarczyk et al., 2016). In addition to the increased scope of the database, we have implemented a new network view that gives the user the ability to view binding affinities of chemicals in the interaction network. This enables the user to get a quick overview of the potential effects of the chemical on its interaction partners.

## Results

### Text mining

**FIGURE 2:**
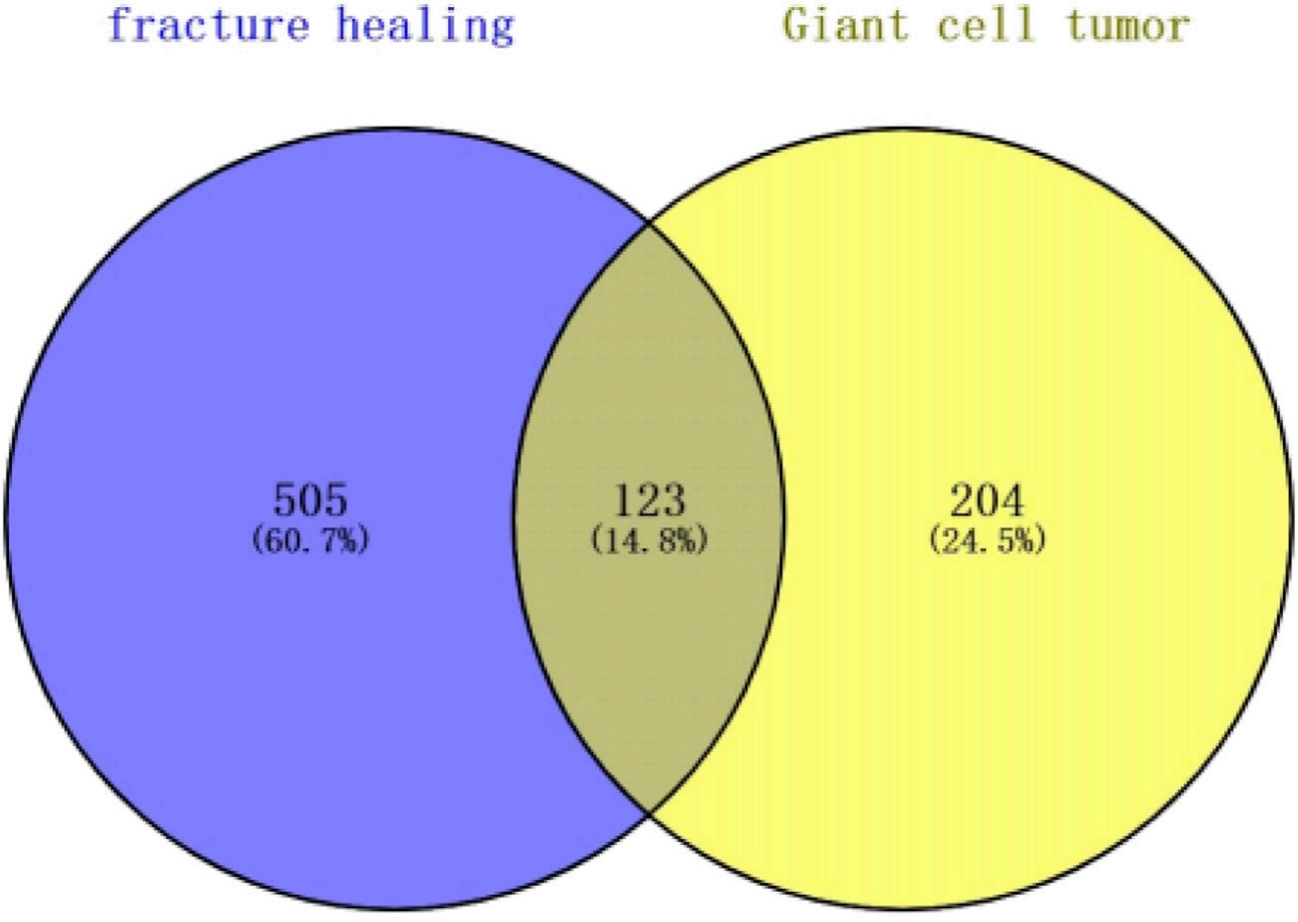
Venn diagram showing the superposed genes between BGCT and BF.

The study is based on the data on the strategy described in Figure 1. We excluded genes with identical names during text mining in pubmed2ensembl in this study. Finally, we determined that BGCT contained 204 genes, bone giant cell tumor 505, and we identified 123 unique genes related to BGCT and FH (Figure 2).

### GO enrichment analysis

The study is based on the characteristics of the results from the analysis of the identified 123 unique genes by GO enrichment annotations in DAVID. The selection of the enriched biological process annotations resulted in six sets of annotations, which were 1) positive regulation of cell proliferation (*p* = 1.50E-34), 2) cell proliferation (*p* = 2.09E-32), 3) response to endogenous stimulus (*p* = 5.42E-32), 4) regulation of cell proliferation (*p* = 3.67E-31), 5) response to oxygen-containing compounds (*p* = 7.10E-31), and 6) response to organic substances (*p* = 1.89E-30) containing 53, 66, 62, 62, 61, and 77 genes from the query set, respectively (Table 2). Repairing fractures is one of the most complex biological processes that occur during human life. After the surgical removal of the bone giant-cell tumors, multiple biological pathways immediately become activated and are synchronized to respond. Hence, the relatively low *p*-value makes these biological process annotations relevant. The cellular component annotations revealed that most of the genes are expressed in the extracellular space, extracellular region part, and extracellular region, and other details are seen in Table 3. The analysis of molecular function annotations resulted in the selection of six pathways. Of the six, the most significant molecular function was receptor binding (*p* = 2.77; E-27), containing 60 genes (Table 4).

**TABLE 2:** Summary of biological process gene set GO enrichment analysis.

Term	Count	*p*-value	Genes
Positive regulation of cell proliferation	53	1.50E-34	*CSF3, PTEN, ILK, PRL, TNF, RPS4X, MYC, CD38, AKT1, TIMP1, IL10, IL11, PDGFRA, EDN1, MMP2, MMP9, RUNX2, SFRP1, IFNG, IL1B, KIT, ANG, CD46, PTH, PDGFB, TNFRSF11A, THBS1, HIF1A, EGFR, PTHLH, INS, NTF3, MAPK1, FGF23, CD55, NTRK2, JUN, TGFB1, VCAM1, EGF, IGF2, FN1, IGF1, ESR1, IL2, VEGFA, IL4, BMP2, IL6, PTPRC, LEP, CTNNB1*, and *FGFR1*
Cell proliferation	66	2.09E-32	*CSF3, SPARC, PTEN, ILK, PRL, NUDT6, TNF, RPS4X, MYC, TIMP2, TNFSF11, CD38, AKT1, TIMP1, CD34, IL10, IL11, PDGFRA, EDN1, HGF, MMP2, MMP9, GTPBP4, RUNX2, SFRP1, IFNG, IL1B, KIT, ANG, CD46, CALCA, PTH, PDGFB, TNFRSF11A, THBS1, HIF1A, EGFR, PTHLH, INS, CD79A, NTF3, CCL2, MAPK1, FGF23, CD55, NTRK1, NTRK2, JUN, TGFB1, ACE, VCAM1, EGF, IGF2, FN1, IGF1, ESR1, IL2, VEGFA, IL4, BMP2, IL6, PTPRC, APC, LEP, CTNNB1*, and *FGFR1*
Response to endogenous stimulus	62	5.42E-32	*SPARC, PTEN, ILK, TNF, MB, MYC, TIMP2, TNFSF10, CD38, AKT1, TIMP1, NOS1, IL10, PDGFRA, EDN1, MMP2, FOS, MMP9, RUNX2, SFRP1, MMP13, IL1B, KIT, ANG, CD44*, *CALCA, PCNA, MAX, PTH, PDGFB, THBS1, EGFR, INS, CNR2, CNR1, BGLAP, NTF3, SPP1, CCL2, MAPK1, FGF23, NTRK1, NTRK2, JUN, TGFB1, ACE, VCAM1, NOS2, IGF2, IGF1, ESR1, POMC, IL4, COL1A1, GH1, BMP2, IL6, COL1A2, APC, LEP, CTNNB1*, and *FGFR1*
Regulation of cell proliferation	62	3.67E-31	*CSF3, SPARC, PTEN, ILK, PRL, NUDT6, TNF, RPS4X, MYC, TIMP2, CD38, AKT1, TIMP1, IL10, IL11, PDGFRA, EDN1, MMP2, MMP9, GTPBP4, RUNX2, SFRP1, IFNG, IL1B, KIT, ANG, CD46, PTH, PDGFB, TNFRSF11A, THBS1, HIF1A, EGFR, PTHLH, INS, NTF3, CCL2, MAPK1, FGF23, CD55, NTRK1, NTRK2, JUN, TGFB1, ACE, VCAM1, NOS2, EGF, IGF2, FN1, IGF1, ESR1, IL2, VEGFA, IL4, BMP2, IL6, PTPRC, APC, LEP, CTNNB1*, and *FGFR1*
Response to oxygen-containing compounds	61	7.10E-31	*CSF3, SPARC, PTEN, TNF, RPS4X, MB, MYC, TNFSF10, CD38, AKT1, TIMP1, NOS1, IL10, PDGFRA, EDN1, HGF, MMP2, FOS, MMP9, RUNX2, SFRP1, MMP13, IL1B, PCNA, MAX, PTH, PDGFB, TNFRSF11A, THBS1, HIF1A, EGFR, INS, MAPK8, CNR2, CNR1, BGLAP, SPP1, CCL2, MAPK1, CD14, FGF23, NTRK1, NTRK2, JUN, TGFB1, ANGPT2, ACE, VCAM1, NOS2, IGF2, IGF1, ESR1, IL2, POMC, COL1A1, GH1, IL6, COL1A2, APC, LEP*, and *CTNNB1*
Response to organic substances	77	1.89E-30	*CSF3, SPARC, PTEN, ILK, TNF, RPS4X, IBSP, MB, MYC, TIMP2, TNFSF10, TNFSF11, CD38, AKT1, TIMP1, NOS1, IL10, PDGFRA, EDN1, HGF, MMP2, FOS, MMP9, RUNX2, SFRP1, MMP13, IFNG, IL1B, KIT, ANG, CD44, CALCA, PCNA, MAX, PTH, PDGFB, TNFRSF11A, THBS1, HIF1A, EGFR, INS, MAPK8, CNR2, CNR1, BGLAP, NTF3, CCL4, SPP1, CCL2, MAPK1, CD14, FGF23, NTRK1, NTRK2, JUN, TGFB1, ANGPT2, ACE, VCAM1, NOS2, IGF2, IGF1, ESR1, IL2, VEGFA, POMC, IL4, COL1A1, GH1, BMP2, IL6, PTPRC*, *COL1A2, APC, LEP, CTNNB1*, and *FGFR1*

**TABLE 3:** Summary of cellular component gene set GO enrichment analysis.

Term	Count	*p*-value	Genes
Extracellular space	60	1.02E-24	*CSF3, SPARC, ITGAM, PRL, SERPINA6, TNF, IBSP, CTSK, TIMP2, TNFSF10, TNFSF11, TIMP1, IL10, IL11, EDN1, HGF, MMP2, MMP9, SFRP1, MMP13, IFNG, IL1B, KIT, ANG, CALCA, PTH, PDGFB, TNFRSF11B, THBS1, EGFR, PTHLH, INS, BGLAP, NTF3, CCL4, SPP1, CCL2, CD14, PROM1, FGF23, TGFB1, ANGPT2, ACE, VCAM1, EGF, IGF2, FN1, IGF1, LYZ, IL2, VEGFA, POMC, IL4, COL1A1, GH1, BMP2, IL6, COL1A2, LEP*, and *ALB*
Extracellular region part	76	7.23E-22	*CSF3, SPARC, ITGAM, PRL, SERPINA6, TNF, RPS4X, IBSP, MB, CTSK, TIMP2, TNFSF10, TNFSF11, CD38, PHGDH, TIMP1, CD34, IL10, IL11, EDN1, MMP1, HGF, MMP2, MMP9, LAT2, SFRP1, MMP13, IFNG, IL1B, KIT, ANG, CD46, CD44, CALCA, PCNA, ABCB6, PTH, PDGFB, TNFRSF11B, THBS1, EGFR, PTHLH, INS, TTN, BGLAP, NTF3, CCL4, SPP1, CCL2, CD14, PROM1, FGF23, CD55, TGFB1, ANGPT2, ACE, VCAM1, EGF, IGF2, FN1, IGF1, LYZ, IL2, VEGFA, POMC, IL4, COL1A1, GH1, BMP2, IL6, DES, PTPRC, COL1A2, LEP, ALB*, and *CTNNB1*
Extracellular region	81	2.42E-20	*CSF3, SPARC, ITGAM, PTEN, PRL, SERPINA6, TNF, RPS4X, IBSP, MB, CTSK, TIMP2, TNFSF10, TNFSF11, CD38, PSMD1, PHGDH, TIMP1, CD34, IL10, IL11, EDN1, MMP1, HGF, MMP2*, *MMP9, LAT2, SFRP1, MMP13, IFNG, IL1B, KIT, ANG, CD46, CD44, CALCA, PCNA, ABCB6, PTH, PDGFB, TNFRSF11B, THBS1, EGFR, PTHLH, INS, TTN, BGLAP, NTF3, CCL4, SPP1, CCL2, MAPK1, CD14, PROM1, FGF23, CD55, TGFB1, ANGPT2, ACE, VCAM1, EGF, IGF2, FN1, IGF1, LYZ, IL2, VEGFA, CD2, POMC, IL4, COL1A1, GH1, BMP2, IL6, DES, PTPRC, COL1A2, LEP, ALB, CTNNB1*, and *FGFR1*
Secretory granule	31	9.30E-14	*SPARC, ITGAM, PDGFB, THBS1, INS, NLRP5, TIMP2, PSMD1, MAPK1, CD38, CD14, TIMP1, CD55, EDN1, TGFB1, EGF, HGF, IGF2, FN1, IGF1, LYZ, MMP9, VEGFA, POMC, COL1A1, PTPRC, IL1B, ALB, KIT, CD46*, and *CD44*
Platelet alpha-granule lumen	12	7.05E-13	*TGFB1, SPARC, EGF, HGF, ALB, PDGFB, IGF2, FN1, TIMP1, IGF1, THBS1*, and *VEGFA*
Vesicle lumen	20	1.29E-12	*CSF3, TGFB1, SPARC, EGF, HGF, PDGFB, IGF2, FN1, IGF1, LYZ, THBS1, EGFR, INS, VEGFA, POMC, ALB, TIMP2, PSMD1, MAPK1*, and *TIMP1*

**TABLE 4:** Summary of molecular function gene set GO enrichment analysis.

Term	Count	*p*-value	Genes
Receptor binding	60	2.77E-27	*CSF3, ITGAM, PTEN, ILK, PRL, TNF, GRIP1, IBSP, PALM, TIMP2, TNFSF10, TNFSF11, TIMP1, IL10, IL11, PDGFRA, EDN1, HGF, SFRP1, IFNG, IL1B, ANG, CALCA, PCNA, PTH, PDGFB, TNFRSF11B, THBS1, HIF1A, EGFR, PTHLH, INS, NTF3, CCL4, SPP1, CCL2, FGF23, NTRK1, TGFB1, ANGPT2, ACE, VCAM1, EGF, IGF2, FN1, IGF1, ESR1, IL2, VEGFA, CD2, POMC, IL4, GH1, BMP2, IL6, PTPRC, LEP, CTNNB1, CD244,* and *FGFR1*
Growth factor activity	18	2.08E-15	*IL10, IL11, CSF3, TGFB1, EGF, HGF, PDGFB, IGF2, IGF1, IL2, VEGFA, IL4, GH1, BMP2, IL6, NTF3, TIMP1*, and *FGF23*
Cytokine activity	20	4.99E-15	*IL10, IL11, CSF3, EDN1, TGFB1, TNFRSF11B, TNF, IL2, VEGFA, IL4, BMP2, IL6, IFNG, IL1B, CCL4, TNFSF10, SPP1, CCL2, TNFSF11*, and *TIMP1*
Cytokine receptor binding	19	1.05E-12	*IL10, NTRK1, IL11, CSF3, TGFB1, PRL, TNF, IL2, VEGFA, IL4, GH1, IL6, IFNG, IL1B, NTF3, CCL4, TNFSF10, CCL2*, and *TNFSF11*
Identical protein binding	43	2.92E-10	*GPSM2, CALCA, PCNA, MAX, PTEN, PDGFB, THBS1, TNF, EGFR, TTN, INS, CD79A, CNR1, CCL4, TNFSF10, AKT1, TNFSF11, MAPK1, CD38, NTRK1, PDGFRA, NTRK2, JUN, TGFB1, NOS2, HGF, FN1, FOS, LYZ, MMP9, ESR1, VEGFA, CD2, COL1A1, SFRP1, DES, COL1A2, SOAT1, ALB, KIT, ALOX5AP, ANG*, and *FGFR1*
Growth factor receptor binding	13	3.85E-10	*IL10, PDGFRA, IL11, CSF3, EGF, PTEN, PDGFB, IL2, VEGFA, IL4, IL6, IL1B,* and *FGF23*

**FIGURE 3:**
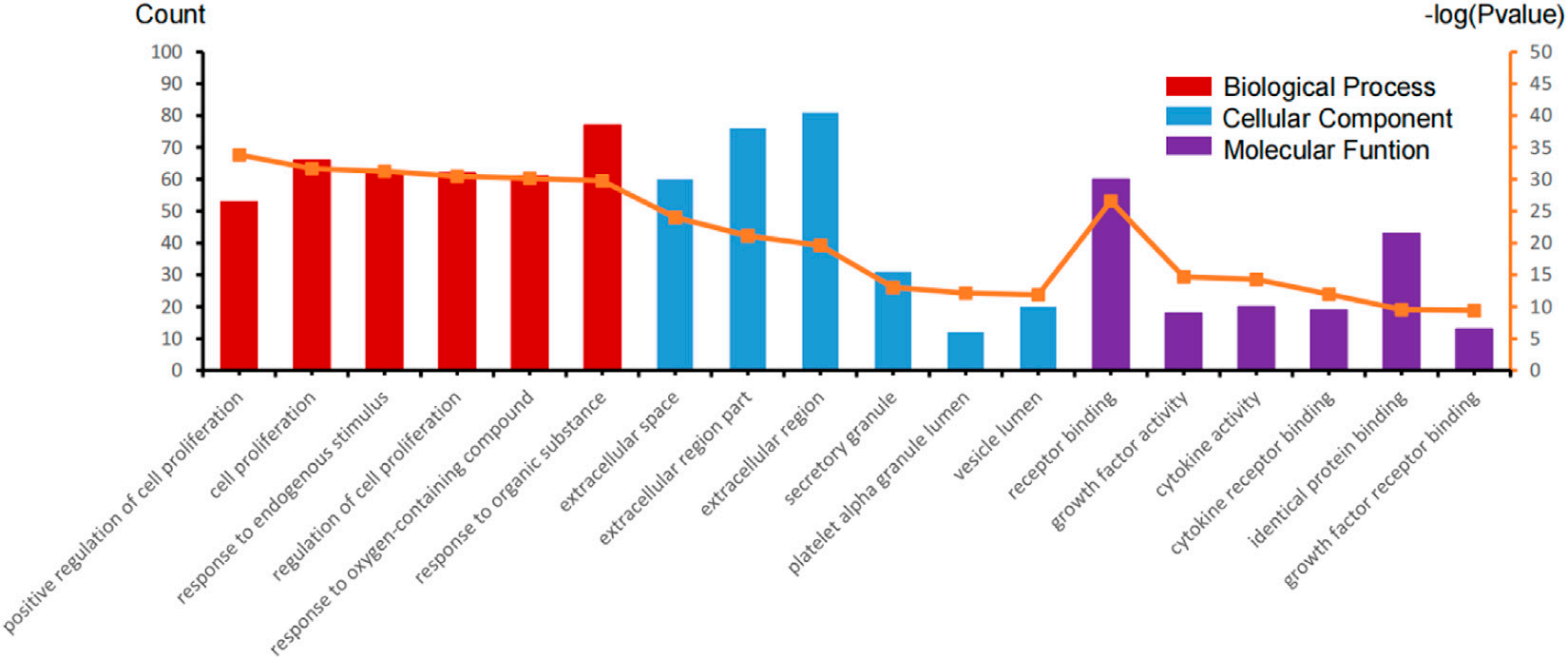
GO enrichment annotation analysis in the biological process (BP), cellular component (CC), and molecular function (MF) of those gene sets.

To more intuitively present GO enrichment annotations details, we conducted mapping with R statistical software (version 3.6.1, Figure 3). A total of 115 genes were screened in the GO enrichment analysis of BP, CC, and MF.

### Kyoto Encyclopedia of Genes and Genomes (KEGG) pathway analysis

**FIGURE 4:**
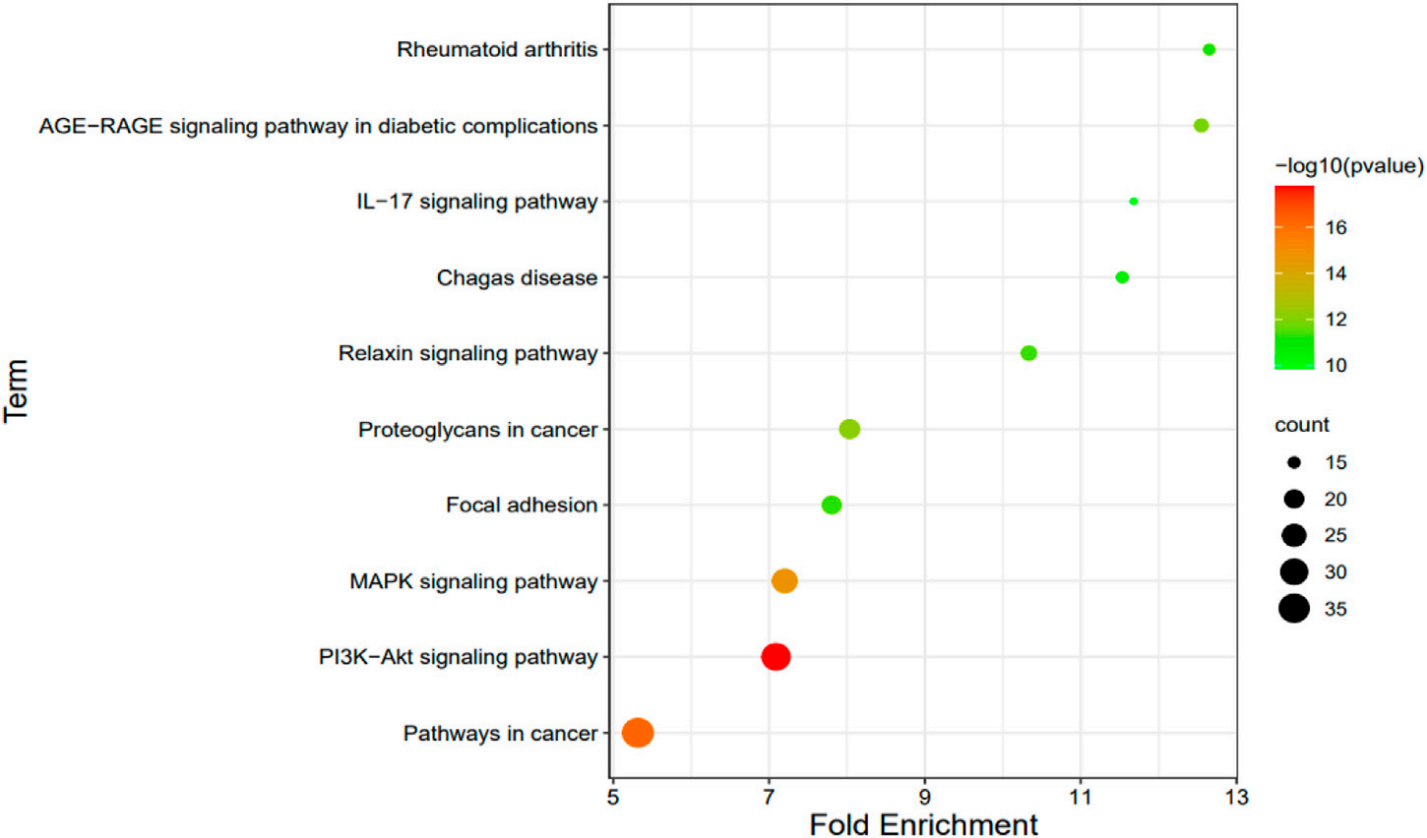
Enriched biological KEGG pathway.

We selected 10 KEGG pathways with lower *p*-values with procedure KEGG pathways enrichment analysis, representing that they were strongly correlated with BGCT and FH (Table 5). The five most enriched biological KEGG pathways were 1) the PI3K-Akt signaling pathway (1.66E-18), 2) pathways in cancer (5.36E-17), 3) MAPK signaling pathway (1.38E-15), 4) proteoglycans in cancer (6.64E-13), and 5) AGE-RAGE signaling pathway in diabetic complications (1.22E-12), containing 32, 36, 27, 21, and 16 genes related to pathway enrichment analysis, respectively, and other highly enriched pathways including the relaxin signaling pathway, focal adhesion, rheumatoid arthritis, Chagas disease, and IL-17 signaling pathway. To bring the details to life, we used R statistics software (version 3.6) for mapping (Figure 4). A total of 68 associated genes were screened out in the 10 KEGG pathways, which will later be used in the protein–protein interaction analysis.

**TABLE 5:** Summary of KEGG pathway enrichment analysis.

Term	Fold Enrichment	*p*-value	Count	Genes
PI3K-Akt signaling pathway	7.089092	1.66E-18	32	*CSF3, PTEN, PDGFB, PRL, THBS1, EGFR, INS, IBSP, MYC, NTF3, SPP1, AKT1, MAPK1, FGF23, NTRK1, PDGFRA, NTRK2, ANGPT2, EGF, HGF, IGF2, FN1, IGF1, IL2, VEGFA, IL4, COL1A1, GH1, IL6, COL1A2, KIT*, and *FGFR1*
Pathways in cancer	5.316819	5.36E-17	36	*MAX, PTEN, PDGFB, HIF1A, EGFR, MAPK8, MYC, AKT1, MAPK1, FGF23, NTRK1, PDGFRA, JUN, EDN1, TGFB1, NOS2, MMP1, EGF*, *HGF*, *MMP2, IGF2, FN1, IGF1, FOS, MMP9, ESR1, IL2, VEGFA, IL4, BMP2, IL6, IFNG, APC, KIT, CTNNB1,* and *FGFR1*
MAPK signaling pathway	7.202119	1.38E-15	27	*MAX, PDGFB, TNF, EGFR, INS, MAPK8, MYC, NTF3, AKT1, MAPK1, CD14, FGF23, NTRK1, PDGFRA, NTRK2, JUN, TGFB1, ANGPT2, EGF, HGF, IGF2, IGF1, FOS, VEGFA, IL1B, KIT,* and *FGFR1*
Proteoglycans in cancer	8.033583	6.64E-13	21	*TGFB1, HGF, MMP2, IGF2, FN1, IGF1, THBS1, HIF1A, ESR1, TNF, MMP9, EGFR, VEGFA, COL1A1, COL1A2, MYC, AKT1, MAPK1, CTNNB1, CD44,* and *FGFR1*
AGE-RAGE signaling pathway in diabetic complications	12.54769	1.22E-12	16	*JUN, EDN1, TGFB1, VCAM1, MMP2, FN1, TNF, VEGFA, COL1A1, IL6, MAPK8, COL1A2, IL1B, CCL2, AKT1*, and *MAPK1*
Relaxin signaling pathway	10.33482	4.21E-12	17	*JUN, EDN1, TGFB1, NOS2, MMP1, MMP2, FOS, MMP9, EGFR, VEGFA, COL1A1, MAPK8, MMP13, COL1A2, AKT1, MAPK1*, and *NOS1*
Focal adhesion	7.803291	4.73E-12	20	*PDGFRA, JUN, EGF, HGF, PTEN, PDGFB, FN1, ILK, IGF1, THBS1, EGFR, VEGFA, COL1A1, MAPK8, COL1A2, IBSP, SPP1, AKT1, MAPK1*, and *CTNNB1*
Rheumatoid arthritis	12.64888	6.93E-12	15	*IL11, JUN, TGFB1, PTH, MMP1, FOS, TNFRSF11A, TNF, VEGFA, IL6, IFNG, IL1B, CTSK, CCL2,* and *TNFSF11*
Chagas disease	11.53281	2.53E-11	15	*IL10, JUN, TGFB1, ACE, NOS2, FOS, TNF, IL2, IL6, MAPK8, IFNG, IL1B, CCL2, AKT1,* and *MAPK1*
IL-17 signaling pathway	11.68003	1.24E-10	14	*CSF3, JUN, MMP1, FOS, TNF, MMP9, IL4, IL6, MAPK8, MMP13, IFNG, IL1B, CCL2,* and *MAPK1*

### Protein–protein interaction (PPI) network and gene module analysis

**FIGURE 5:**
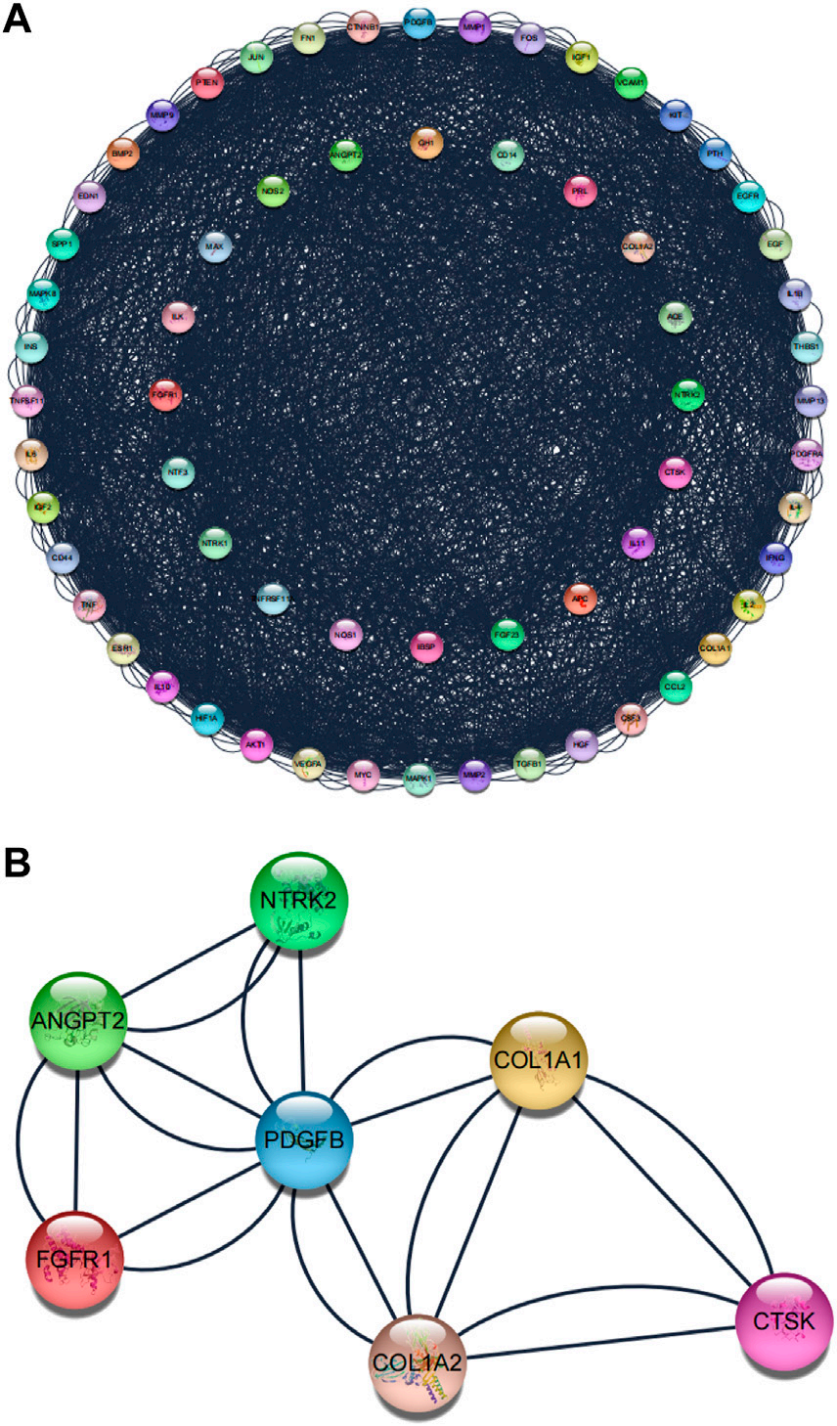
Protein–protein interaction (PPI) network. **(A)** Protein–protein interaction (PPI) network of the superposed genes between BGCT and BF. **(B)** Tightest module from the PPI network.

The Protein–protein interaction analysis (PPI) was performed using the STRING database. This study set the following parameters minimum required interaction score: highest confidence (0.900) and hidden nodes in the network. Network stats showed the following results: the number of nodes: 64, the number of edges: 2248, average node degree: 6.94, local clustering coefficient: 0.509, network centralization: 0.358, and PPI enrichment *p*-value: <1.0e-16 (Figure 5A). After that, Cytoscape software constructed PPI networks. The significant gene modules of the PPI networks were applied to pick out in Cytoscape with the Molecular Complex Detection (MCODE). The parameters were set as follows: the degree cutoff >2, K-scores >2, and node score cutoff >0.2. Finally, based on these criteria, we selected seven central genes, which formed the tightest module network, including *ANGPT2*, *COL1A1*, *COL1A2*, *CTSK*, *FGFR1*, *NTRK2*, and *PDGFB* (Figure 5B).

#### The hub genes’ qRT-PCR validation

**FIGURE 6:**
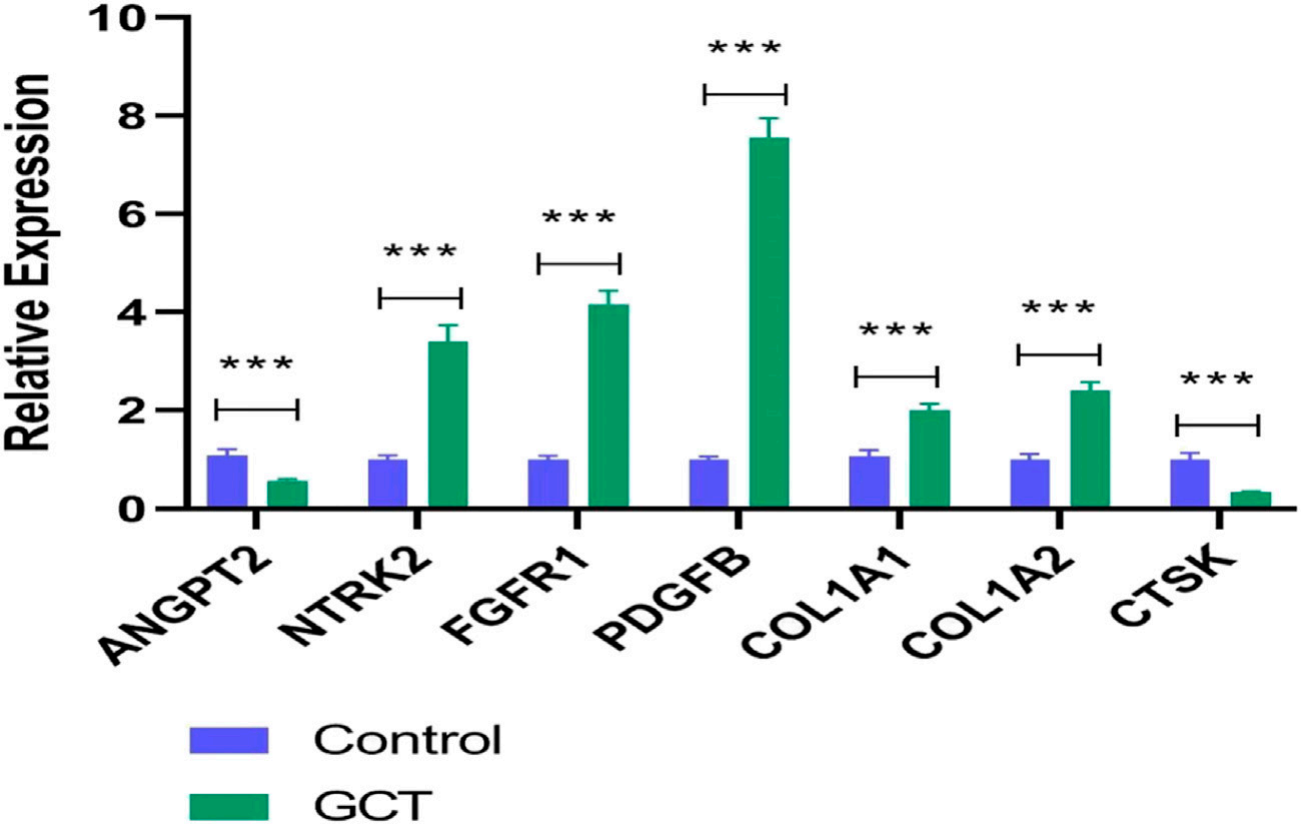
Interrelation of 17 drugs with genes and pathways.

A qRT-PCR approach was used to detect the expression levels of seven potential genes. The verification result showed that the expression levels of *NTRK2*, *FGFR1*, *PDGFB*, *COL1A1*, and *COL1A2* were significantly increased in gct samples (*p* < 0.05) (Figure 6), which confirmed the analytical signaling pathway results of bioinformatics were reliable in this study.

### Drug–gene interactions

**FIGURE 7:**
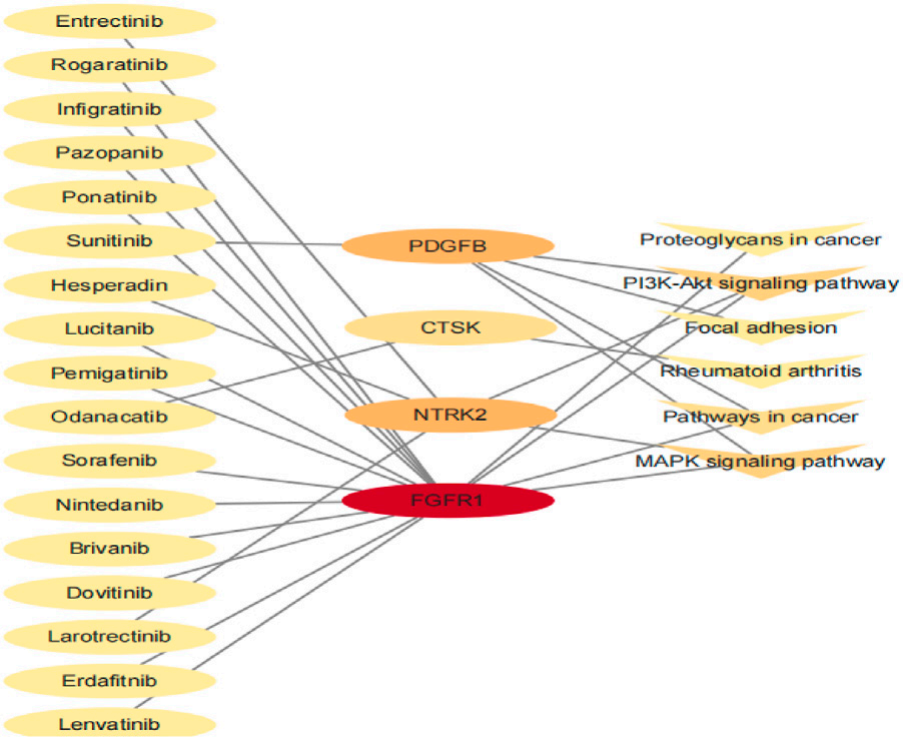
Interrelation of 17 drugs with genes and pathways.

Finally, we conducted a drug–gene interaction analysis on the seven genes screened by the Module, and 144 drugs had specific effects on the aforementioned genes and pathways. The potential gene targets were *ANGPT2* (4 drugs), *COL1A1*, *COL1A2* (2 drugs each), *CTSK* (5 drugs), *FGFR1* (85 drugs), *NTRK2* (42 drugs), and *PDGFB* (4 drugs). In this study, we selected the drug with PMIDs, and the drug–gene interaction defined that it has been studied to some extent. A list of 17 drugs thought to potentially treat bone giant cell tumors is given in Table 6. There were 15 antineoplastic drugs, 1 antiosteoporosis drug, and 1 anti-influenza drug. The relationship between the genes and pathways corresponding to the 17 drugs is shown in Figure 7.

**TABLE 6:** Candidate drugs targeting genes with giant cell tumor (GCT).

Number	Drug	Description	Gene	Drug–gene interaction	Score	Approved by FDA	Reference (PubMed ID)
1	Odanacatib	Antiosteoporotic agent	CTSK	Inhibitor	24.73	No	18226527
2	Lucitanib	Antineoplastic	FGFR1	Inhibitor	0.73	No	27126994 and 25193991
3	Erdafitinib	Antineoplastic	FGFR1	Inhibitor	0.73	No	28341788 and 26324363
							28965185
4	Rogaratinib	Antineoplastic	FGFR1	Inhibitor	0.58	No	30807645
5	Pemigatinib	Antineoplastic	FGFR1	Inhibitor	0.48	Yes	32315352
6	Infigratinib	Antineoplastic	FGFR1	Inhibitor	0.43	Yes	22837287 and 26015511
							27535980
7	Ponatinib	Antineoplastic	FGFR1	Inhibitor	0.33	No	22238266 and 23563700
							26175911 and 26179511
							24771645 and 23468082
8	Brivanib	Antineoplastic	FGFR1	Inhibitor	0.33	No	22238366 and 20124951
9	Nintedanib	Antineoplastic	FGFR1	Inhibitor	0.31	Yes	22238366 and 18559524
							31016670
10	Lenvatinib	Antineoplastic	FGFR1	Inhibitor	0.24	No	25295214 and 17943726
11	Dovitinib	Antineoplastic	FGFR1	Inhibitor	0.08	No	22238366 and 23658459
							27315356 and 17698633
12	Sorafenib	Antineoplastic	FGFR1	Inhibitor	0.06	Yes	25900027 and 17016424
		immunotherapy					28362716 and 15466206
							16507829
13	Pazopanib	Antineoplastic	FGFR1	Inhibitor	0.05	Yes	24302556
		immunotherapy					
14	Larotrectinib	Antineoplastic	NTRK2	Inhibitor	2.58	No	32315394 and 29606586
							29920189
15	Entrectinib	Antineoplastic	NTRK2	Inhibitor	0.22	No	26939704 and 32315394
							30425456 and 30050303
							26457764
16	Hesperadin	Influenza antiviral agent	NTRK2	Inhibitor	0.04	No	19035792
17	Sunitinib	Antineoplastic	PDGFB	Inhibitor	1.19	Yes	29760553
		immunotherapy					

## Discussion

The classic treatment of bone giant cell tumors is still local curettage plus postoperative denosumab treatment. However, the current literature shows that the long-term use of denosumab may increase the risk of local recurrence and sometimes lead to side effects such as arthralgias, muscle pain, hypophosphatemia, and hypercalcemia (Luengo-Alonso et al., 2019). The elucidation of the pathophysiology of giant cell tumors of bone, particularly regarding the role of the nuclear factor κ B ligand (RANKL), led to the approval of denosumab by the US Food and Drug Administration (FDA) for the treatment of locally advanced or metastatic GCTB. The treatment paradigm has shifted from local to multidisciplinary treatment, considering denosumab in advanced giant cell tumors where surgical resection alone can lead to severe morbidity (Basu Mallick and Chawla, 2021). A growing number of studies have recently suggested that denosumab may increase the risk of local recurrence in patients undergoing curettage. It may be due to the thickening of the bone margin of the tumor, which intercepts tumor cells during scraping. After denosumab treatment, direct osteogenesis of marginal tumor cells also leads to local recurrence. *In vitro* studies have shown that denosumab produces a cytostatic response rather than an accurate cytotoxic response to tumor stromal cells (Li et al., 2020). In order to solve this problem, we use bioinformatics tools to identify existing potential fedinomab drugs for the treatment of bone giant cell tumors. Therefore, we identified 4 potential targeting genes and 13 drugs associated with bone giant cell tumors in Table 6.

Histologically, bone giant cell tumors consist of neoplastic spindle-shaped stromal cells, large multinucleated osteoclast-like cells, and their monocytic precursors expressing the corresponding receptor. Cathepsin K and its related transmembrane proton pump V-ATPase are significantly expressed in osteoclast giant cells, which are the main proteolytic enzymes in BGCT and participate in the degradation of bone collagen matrix in the metaphysis of BGCT (Lindeman et al., 2004). In addition, cathepsin K also plays a key role in bone homeostasis; low expression is related to bone resorption damage, and high expression is related to bone loss (Stroup et al., 2001). The mechanism of bone giant cell tumor at the pathological and genetic levels involves many factors. The fibroblast growth factor (FGF) is the key to tumor survival, migration, and tumor angiogenesis. It is overexpressed in a variety of cancers (glioblastoma, lung, gastric cancer, liver, endometrium, and urothelial carcinoma). Mutations of the fibroblast growth factor receptor type 1 (FGFR1) genes are one of the characteristic molecular changes of giant-cell-rich bone tumors (Hartmann et al., 2021). Hasenfratz et al. (2021) reported three cases of denosumab. After treatment, one of the BGCT patients with malignant H3F3A mutation developed to a pleomorphic sarcoma. The results of gene sequencing showed that the H3F3A mutation had been lost in the original bone giant cell tumor, but the FGFR1 mutation still existed. Related findings suggest that the H3F3A mutation analysis is a particular, although less sensitive, diagnostic tool for differentiating GCTB and chondroblastoma-forming tumors from other giant cell tumors (Cleven et al., 2015). In the World Health Organization Classification of Bone and Soft Tissue Tumors, NTRK fusions in sarcomas account for an increasing proportion (Gatalica et al., 2019). Neurotrophic tyrosine kinase 2 (NTRK 2) is a member of the family of receptor tyrosine kinases, which is mainly involved in the development of nerve tissue, differentiation, and metabolism. It has a high affinity for nerve growth factor receptor and can activate MAP kinase and the PIK3CA downstream pathway (Chen et al., 2021). PDGFB-COL1A1 gene fusion is considered to be related to giant-cell fibroblastoma, dermatofibrosarcoma protuberans, and other bone and soft tissue tumors (Li et al., 2018). Zhu and Qiu (1994) carried out a cytogenetic study on 19 cases of bone giant cell tumor and 4 cases of BGCT. It was found that the 22q12 locus was homologous to or adjacent to oncogene PDGF B (sis).

In our study, the drugs responsible for the inhibitor the FGFR1 gene corresponded to the largest number of drug categories (nasty 13). Although these drugs have not been applied for the treatment of BGCT, they have been approved by the FDA for the treatment of other diseases with excellent efficacy. Erdafitinib (JNJ-42756493), an oral pan-FGFR tyrosine kinase inhibitor, has been shown to have targeted regulatory effects on aberrant ligand-dependent FGFR signaling and activated cellular models of non-small cell lung, breast, bladder, endometrial, gastric, and colon cancer, as well as certain hematological malignancies in cytological experiments. In the mouse model of xenotransplantation of FGFR-related stomach, bladder, and squamous NSCLC tumors, erdafitinib also showed strong antitumor activity (Perera et al., 2017). In a multicenter, open-label phase I human trial, erdafitinib administered at 10 mg on a 7-day-on/7-day-off schedule was able to obtain clinical responses and controllable side effects. A phase I human trial in Japan also showed that erdafitinib was well tolerated in patients with a variety of advanced or refractory solid tumors (Nishina et al., 2018).

## Conclusion

The 7 genes (including *ANGPT2*, *COL1A1*, *COL1A2*, CTSK, FGFR1, NTRK2, and PDGFB) and 17 drugs, which have not been used in BGCT, but 6 drugs approved by the FDA for other diseases, could be potential genes and drugs, respectively, to improve BGCT treatment. In addition, the correlation study and analysis of potential drugs through genes provide great opportunities to promote the repositioning of drugs and the study of pharmacology in the pharmaceutical industry.

## Data Availability

The original contributions presented in the study are included in the article/supplementary material; further inquiries can be directed to the corresponding authors.
